# Improvement in ADLs during a Nursing Home Stay for Older Adults with Stroke, Joint Replacement or Hip Fracture

**DOI:** 10.1093/geroni/igaa057.3354

**Published:** 2020-12-16

**Authors:** Warona Mathuba, Brian Downer, Rachel Deer

**Affiliations:** The University of Texas Medical Branch at Galveston, Galveston, Texas, United States

## Abstract

Regularly assessing the health and function of older adults who are in the hospital is important for preventing poor outcomes. Such information may also be useful in post-acute care settings, such as skilled nursing facilities (SNFs) to identify older adults who are high risk for poor outcomes. This study had two objectives: Map items from the Acute Care for the Elderly (ACE) Tracker to items from the Minimum Data Set (MDS). (2) Examine the association between ACE Tracker items and improvement in activities of daily living (ADLs) during a SNF stay. We identified Medicare fee-for-service beneficiaries admitted to a SNF within 3 days of hospital discharge for a hip fracture (n=118,790), joint replacement (n=245,845), or stroke (n=64,153). Items from the ACE Tracker were matched to patients’ first MDS assessment. The first and last MDS assessments were used to calculate a total score for self-performance on seven ADLs. Multivariable logistic regression models were used to identify patient characteristics associated with the odds for improvement in ADL function. Severe ADL limitations at admission and greater hours of physical and occupational therapy were associated with significantly higher odds of ADL improvement. Cognitive impairment, vision limitations, indwelling catheters, and unhealed pressure ulcers were associated with significantly lower odds of ADL improvement. The characteristics associated with improved ADL function were similar between patients with joint replacement, hip fracture, and stroke. Many of the health and functional characteristics routinely measured in hospital settings are also collected in SNFs and are associated with improvement in ADL function.

